# Urinary Biomarkers of Acute Kidney Injury in Patients with Liver Cirrhosis

**DOI:** 10.1155/2014/376795

**Published:** 2014-04-06

**Authors:** Anass Ahmed Qasem, Salama Elsayed Farag, Emad Hamed, Mohamed Emara, Ahmed Bihery, Heba Pasha

**Affiliations:** ^1^Internal Medicine Department, Faculty of Medicine, Zagazig University, Zagazig 44511, Egypt; ^2^Tropical Medicine Department, Faculty of Medicine, Zagazig University, Zagazig 44511, Egypt; ^3^Medical Biochemistry Department, Faculty of Medicine, Zagazig University, Zagazig 44511, Egypt

## Abstract

Acute kidney injury (AKI) is a common complication in cirrhotic patients. Serum creatinine is a poor biomarker for detection of renal impairment in cirrhotic patients. This study aimed to evaluate urinary neutrophil gelatinase-associated lipocalin (NGAL) and urinary interleukin-18 (IL-18) as early biomarkers of acute kidney injury in cirrhotic patients. 160 patients with cirrhosis admitted to the Liver Units at Zagazig University Hospitals were classified into three groups: (I) nonascitic patients, (II) ascitic patients without renal impairment, and (III) ascitic patients with renal impairment. Patients with renal impairment were further divided into four subgroups: [A] prerenal azotemia, [B] chronic kidney disease (CKD), [C] hepatorenal syndrome (HRS), and [D] acute tubular necrosis (ATN). Significant elevation of both urinary NGAL and urinary IL-18 in cirrhotic patients with renal impairment especially in patients with ATN was observed. Urinary NGAL and urinary IL-18 have the ability to differentiate between AKI types in patients with cirrhosis. This could improve risk stratification for patients admitted to the hospital with cirrhosis, perhaps leading to early ICU admission, transplant evaluation, and prompt initiation of HRS therapy and early management of AKI.

## 1. Introduction

Egypt was blessed with the River Nile and ancient culture but was deemed with liver diseases. Liver cirrhosis is a common disease in Egypt as Egypt has the highest incidence rate of HCV infection worldwide [1].

Acute kidney injury (AKI) in patients with cirrhosis is common. Up to 20% of hospitalized patients with cirrhosis develop AKI [2] and once AKI occurs, there is a reported fourfold increased risk of mortality [3].

Typically, patients with decompensated liver cirrhosis have significant circulatory dysfunction, which is characterized by a vasodilatory state, lower total peripheral resistance, activated renin-angiotensin-aldosterone system (RAAS), and finally renal arterial vasoconstriction [4].

In cirrhosis, AKI types include prerenal azotemia, hepatorenal syndrome (HRS), and acute tubular necrosis (ATN) with prevalence rates of 68%, 25%, and 33%, respectively, but their effect on mortality risk varies [2]. Unfortunately, these forms of AKI are difficult to distinguish clinically as serum creatinine (sCr), the clinical standard to define kidney function, poorly discriminates AKI type in cirrhosis [5]. Furthermore, various factors can affect serum creatinine in cirrhotic patients such as age, gender, nutritional status, muscle mass, and drug and volume distribution; in addition, deranged hepatic synthesis of the creatine may contribute to impaired creatinine production in liver cirrhosis so utilization of serum creatinine concentration for diagnosing renal dysfunction could be even more unsatisfactory [4].

Recently, in an effort to improve the definition of AKI and to highlight the importance of non-HRS kidney dysfunction in cirrhosis, the Acute Dialysis Quality Initiative (ADQI) and the International Ascites Club (IAC) jointly published a consensus statement regarding AKI classification [6], incorporating the Risk, Injury, Failure, Loss, and End-Stage Disease (RIFLE) and the Acute Kidney Injury Network (AKIN) guidelines. IAC definition of HRS [7] was classified as a specific form of AKI [8].

Management of AKI in the setting of cirrhotic patients depends primarily on the detection of the cause. HRS is treated pharmacologically whereas prerenal azotemia is treated with plasma volume expansion which is not suitable in the management of ATN [9, 10].

Neutrophil-gelatinase-associated lipocalin (NGAL) is a novel biomarker for diagnosing acute kidney injury (AKI). Several studies have demonstrated the utility of early NGAL measurements for predicting the severity and clinical outcomes of AKI [11–14].

In fact, urine interleukin-18 (IL-18) has been shown to serve as an accurate biomarker to differentiate ATN from other etiological factors of renal disease [15]. Moreover, the prognostic role of urine IL-18 has been validated in general patient groups admitted to ICU [16].

Therefore, this study was to assess the ability of urinary NGAL and IL-18 as early biomarkers of AKI in patients with cirrhosis.

## 2. Patients and Methods

### 2.1. Study Protocol

This is a cross-sectional study including 160 patients with cirrhosis admitted to the Liver Units at Zagazig University Hospitals from July 2012 to December 2012 with history of follow-up in outpatient clinics. The majority of patients were hospitalized for treatment of complications of cirrhosis. Exclusion criteria include (1) patients with hepatocellular carcinoma or cholangiocarcinoma, (2) liver transplant patients, (3) chronic kidney diseases patients maintained on regular hemodialysis before admission, and (4) kidney transplant patients.

The study design was approved by an Institutional Review Board (IRB) of Faculty of Medicine, Zagazig University. Patients included in the study gave informed written consent in accordance with the Declaration of Helsinki.

Clinical and biochemical data were collected at the time of admission to Liver Units at Zagazig University Hospitals with reference to previous patients' data done in outpatient clinics. Child-Pugh score and MELD score were calculated for admission [17, 18]. Estimated GFR was calculated using MDRD equation [19]. A fresh urine sample was taken to measure the urinary levels of sodium, creatinine, NGAL, and IL-18. Venous blood samples were drawn from all participants, and sera were separated immediately.

### 2.2. Classification of Patients

Patients were classified into three groups: (I) nonascitic patients (*n* = 42), (II) ascitic patients without renal impairment (*n* = 50), and (III) ascitic patients with renal impairment (*n* = 68).

This classification was used because it reflects the different stages of cirrhosis [20]. Patients with renal impairment were further divided into four subgroups: [A] prerenal azotemia, [B] chronic kidney disease (CKD) [21], [C] HRS [7], and [D] ATN [22]. The definitions for these different causes of renal impairment are shown in Section 5.

### 2.3. Analytic Procedures

Urine samples for NGAL and IL-18 levels were immediately centrifuged, separated, and stored at −80°C until further analysis. Urinary NGAL was measured using a NGAL ELISA kit (BioVendor GmbH, Germany) in relation to urinary creatinine. Urinary IL-18 was measured using a human IL-18 enzyme-linked immunosorbent assay kit (Medical and Biologic Laboratory, Nagoya, Japan) in relation to urinary creatinine.

Routine biochemical parameters were measured by colorimetric methods (Spinreact, SA Ctra, and Santa Coloma, Spain).

### 2.4. Statistical Analysis

Results are expressed as means ± SD (standard deviation) for continuous variables, while Counts and percentages for categorical variables. Analysis of variance (ANOVA) was used to examine the difference between means of continuous normal-distributed variables.and Chi-square test was used to compare categorical variables. Correlations were evaluated by nonparametric Spearman's correlation. The significance level for all statistical two-tailed tests were accepted as *P* < 0.05. The statistical analyses were conducted using SPSS for Windows (version 19.0; SPSS Inc., Chicago, IL, USA).

## 3. Results

### 3.1. Characteristics of the Patient Population

The demographic, clinical data and biochemical parameters of all patients are shown in Table 1. Patients with renal impairment were divided into the four subgroups as shown in Table 2.

### 3.2. Urinary Neutrophil-Gelatinase-Associated Lipocalin (NGAL)

Patients with renal impairment had higher urinary NGAL levels (357.78 ± 228.51 *μ*g/g creatinine) compared to those of patients without renal impairment, either with or without ascites (96.84 ± 35.58, 113.76 ± 47.98 *μ*g/g creatinine, resp.).

**Figure 1:**
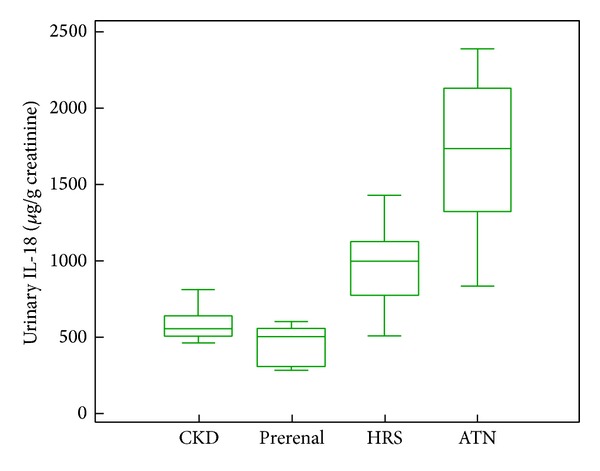
Box-plot of urinary NGAL levels, according to the study subgroups of impairment of kidney function.

The mean value of urinary NGAL in HRS (380.6 ± 132.32 *μ*g/g creatinine) was significantly higher compared to prerenal azotemia patients (161.15 ± 60.75 *μ*g/g creatinine, *P* = 0.0015) and significantly lower compared to ATN patients (580.51 ± 238.75, *P* = 0.0001). Furthermore, urinary NGAL levels in patients with CKD (232.63 ± 41.31 *μ*g/g creatinine) were significantly different from those of patients with HRS (*P* = 0.003) (Figure 1).

In simple regression analysis, urinary NGAL was positively correlated with serum creatinine (*r* = 0.465, *P* < 0.001), urinary IL-18 (*r* = 0.9, *P* < 0.001), fractional excretion of Na (FeNa) (*r* = 0.687, *P* < 0.001), and mean blood pressure (*r* = 0.339, *P* = 0.005) in patient subgroups with renal impairment (Table 3).

The cut-off value of urinary NGAL that differentiates between patients with AKI and those with other causes of renal impairment was 286.3 *μ*g/g creatinine (area under ROC curve is 0.909) (sensitivity 95.5% and specificity 76.1%) with a positive predictive value (PPV) of 65.6 and negative predictive value (NPV) of 99.2 (Figure 3).

### 3.3. Urinary Interleukin-18 (IL-18)

Patients with renal impairment had higher urinary IL-18 levels (983 ± 594 *μ*g/g creatinine) compared to those of patients without renal impairment, either with or without ascites (296.56 ± 113, 254.5 ± 77.9 *μ*g/g creatinine, resp.).

**Figure 2:**
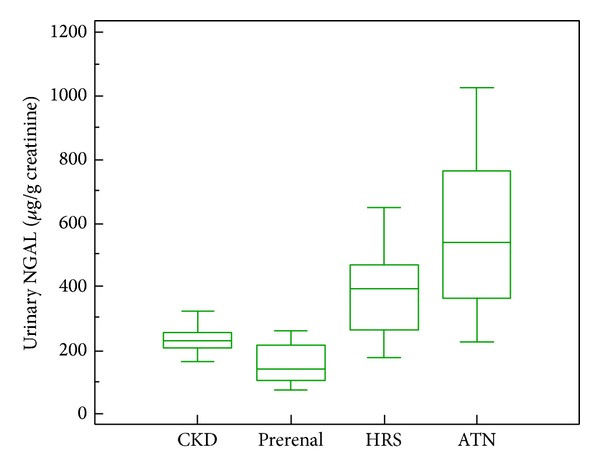
Box-plot of urinary IL-18 levels, according to the study subgroups of impairment of kidney function.

Urinary IL-18 levels were analyzed in renal impairment patients' subgroups. We observed that patients with ATN had the highest values of urinary IL-18, while patients with prerenal azotemia had the lowest values (1687 ± 447 versus 451.47 ± 121.73 *μ*g/g creatinine, resp.; *P* = 0.0001). Patients with HRS had intermediate values (953 ± 273 *μ*g/g creatinine), which were significantly higher than those of patients with prerenal azotemia (*P* = 0.0015) and lower than those of patients with ATN (*P* = 0.0001). Urinary IL-18 levels in patients with CKD (582 ± 98.24 *μ*g/g creatinine) were significantly different from those of patients with HRS (*P* = 0.001) (Figure 2).

In simple regression analysis, urinary IL-18 was positively correlated with serum creatinine (*r* = 0.422, *P* < 0.001), FeNa (*r* = 0.807, *P* < 0.001), and mean blood pressure (*r* = 0.329, *P* = 0.006) in patient subgroups with renal impairment (Table 3).

**Figure 3:**
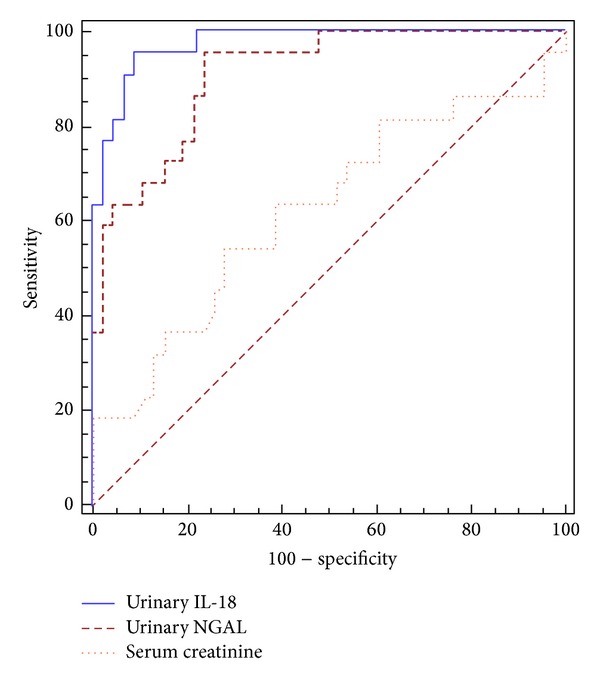
Receiver operating characteristic (ROC) curves to evaluate the capability of urinary IL-18, urinary NGAL, and serum creatinine to diagnose acute tubular injury.

The cut-off value of urinary IL-18 that differentiates between patients with AKI and those with other causes of renal impairment was 1119.6 *μ*g/g creatinine (area under ROC curve is 0.975) (sensitivity 95.5% and specificity 91.3%) with PPV of 84 and NPV of 99.3 (Figure 3).

## 4. Discussion

AKI is a common condition in cirrhotic patients admitted to ICU. It is now recognized that AKI can significantly impact patients' outcomes [21]. In fact, ATN is an important precipitating factor for the development of HRS [2, 18]. It has been proposed that intense renal vasoconstriction in HRS, if prolonged, may lead to tubular ischemia and ultimately progress into ATN [23–25]. Therefore, it may be difficult to distinguish HRS from ATN in clinical settings.

In general, patients without liver cirrhosis, urine sediment and FeNa may help make the diagnosis of ATN. However, confounding results may be encountered in cirrhotic patients that may not correspond to the usual range for general patients with ATN. Therefore, FeNa is a less useful diagnostic tool for tubular injury in patients with advanced liver diseases, whose pre-existing hemodynamic states make them sodium-avid [26, 27].

Our study demonstrated a significant difference of urinary NGAL and IL-18 in each category of AKI: highest in ATN, intermediate in HRS, and low in prerenal disease. Moreover, urinary NGAL and IL-18 levels in patients with prerenal azotemia were similar to those with normal kidney function and stable CKD. In contrast, sCr was not different between patients with ATN and HRS.

The mechanism by which patients with HRS have intermediate urinary NGAL and IL-18 levels remains unclear. HRS physiology is thought to be an extreme prerenal state [27, 28] with severe renovascular vasoconstriction and decreased GFR, but normal intrinsic kidney function. Kidney function can return to normal after improvement of hepatic hemodynamics [27–29] or after renal transplantation into a recipient with normal hepatic function [30, 31].

However, pathologic investigations have reported subtle kidney tubular and glomerular damage in HRS kidneys, some seen only by electron microscopy [23, 32], perhaps resulting from the cellular changes associated with chronic activation of angiotensin-aldosterone signaling [33]. It is conceivable that profound renovascular constriction may cause subclinical tubular damage in at least a subset of nephrons, not detectable by urinary sodium, which is not sensitive enough to detect mild or patchy tubular epithelial damage.

Our study also confirms previous studies demonstrating that a single urinary NGAL or urinary IL-18 measurement on hospital admission has the potential to assist in determining the type of kidney dysfunction, perhaps improving patient management and outcomes [13, 34, 35].

Urinary NGAL and urinary IL-18 demonstrated an excellent discriminating power compared to serum creatinine (AUROC 0.909 for NGAL, 0.975 for IL-18, and 0.622 for Cr) for discriminating ATN from HRS in our patients. This is in contrast to traditional measurements of kidney and liver disease severity including sCr and MELD score.

Our data demonstrate that patients with cirrhosis are at a high risk for acute renal impairment (33.1% of the patients, 41% of them diagnosed as ATN and 26.4% diagnosed as HRS). The present findings seem to be consistent with previous studies [3, 36] which confirm that non-HRS types of AKI are common in patients with cirrhosis and further studies are needed to determine inciting factors of AKI in this population.

This study has some limitations. First, sCr inaccurately measures kidney function in cirrhosis [5, 37, 38], and there is no widely available technique to accurately measure GFR in these patients. Second, absence of kidney biopsy, which is rarely performed in this population, because of the high risk of bleeding tendency, therefore, there is a lack of correlation between renal pathology and urinary NGAL and urinary IL-18.

In conclusion, urinary NGAL and urinary IL-18 have the ability to differentiate between AKI types in patients with cirrhosis. This could improve risk stratification for patients admitted to the hospital with cirrhosis, perhaps leading to early ICU admission, transplant evaluation, and prompt initiation of HRS therapy and early management of AKI. These findings, if confirmed in larger cohorts, could lead to the development of biomarker algorithms to rapidly identify patients with HRS and ATN and accurately predict prognosis.

## 5. Definitions

### 5.1. Cirrhosis

The diagnosis of cirrhosis was based on a combination of clinical, biochemical, ultrasonographic, and endoscopic findings.

### 5.2. Impairment of Kidney Function

Impairment of kidney function was diagnosed when serum creatinine concentration was greater than 1.5 mg/dL. This value of serum creatinine was chosen because it has been selected in several consensus conferences as cut-off to define impairment of kidney function in cirrhosis [7, 20].

### 5.3. Causes of Impairment of Kidney Function

The definitions used for the different causes of impairment of kidney function were as follows. Briefly, (1) prerenal azotemia due to volume depletion was considered when patients had a history of fluid losses in the preceding days (due to either bleeding, diuretic overdose, or other causes), together with compatible findings, absence of other causes of impairment of kidney function, and reversibility of kidney impairment as indicated by decrease of serum creatinine below 1.5 mg/dL after fluid resuscitation; (2) chronic kidney disease (CKD) was defined by evidence of structural kidney abnormalities (imaging studies), proteinuria, and/or abnormal urinalysis, with a glomerular filtration rate of less than 60 mL/min per 1.73 m2, as assessed by MDRD formula [38]; (3) HRS was defined using the current definition of the International Ascites Club [7]; and (4) ATN was diagnosed in patients who had at least three of the following: hypovolemic and/or septic shock or treatment with potentially nephrotoxic agents, urine sodium greater than 40 mEq/L, urine osmolality lower than 400 mOsm/kg, and fractional excretion of sodium greater than 2% without diuretics [22, 39].

## Figures and Tables

**Table 1:** Demographic, clinical, and laboratory data of all patients.

	No ascites (*n* = 42)	Ascites without impairment of kidney function (*n* = 50)	Ascites with impairment of kidney function (*n* = 68)	*P*
Age (years)	53.3 ± 9.6	54.04 ± 11.03	52.95 ± 9.74	0.85
Gender, male	24 (57%)	30 (60%)	49 (72%)	0.21
Etiology of cirrhosis HCV/HBV/others	32/7/3	39/8/3	54/10/4	0.99
Hepatic encephalopathy, *n* (%)	6 (14%)	19 (38%)	32 (47%)	0.004
Gastrointestinal bleeding, *n* (%)	7 (16%)	13 (26%)	25 (36%)	0.173
Serum bilirubin (mg/dL)	2.8 ± 1.76	4.4 ± 2.6	6.9±4	0.0001
Serum albumin (g/L)	31.5 ± 6.1	25.58 ± 3.69	27.72 ± 5.61	0.0001
Prothrombin time (%)	67.14 ± 18.4	58.18 ± 8.84	47.91 ± 15.92	0.0001
Serum creatinine (mg/dL)	0.761 ± 0.19	1.045 ± 0.26	2.38 ± 0.95	0.0001
Glomerular filtration rate (mL/min/1.73 m^2^)	119.53 ± 33.57	85 ± 21.87	45.18 ± 21.28	0.0001
Serum sodium (mEq/L)	135.26 ± 3.8	134.92 ± 4.96	131.72 ± 6.46	0.001
Serum potassium (mEq/L)	3.979 ± 0.42	4 ± 0.54	4.39 ± 0.78	0.001
Child-Pugh score	7.48 ± 1.86	9.86 ± 1.22	10 ± 2	0.0001
MELD score	13.54 ± 3.06	16. ± 4.81	25.74 ± 7.76	0.0001
Mean arterial pressure (mmHg)	83.76 ± 13.9	82.66 ± 15.5	77 ± 14.8	0.035
Urine sodium (mEq/L)	49.71 ± 34.5	47.34 ± 29.34	36.6 ± 21.27	0.029
Urinary IL-18 (µg/g creatinine)	254.5 ± 77.9	296.56 ± 113	983.48 ± 594.43	0.001
Urinary NGAL (µg/g creatinine)	96.84 ± 35.58	113.76 ± 47.98	357.78 ± 228.51	0.001

MELD: Model for End-Stage Liver Disease and NGAL: neutrophil-gelatinase-associated lipocalin.

Significant at *P* < 0.05; *P* < 0.01; and *P* < 0.001.

**Table 2:** Characteristics of renal impairment patients.

	CKD (*n* = 15)	Prerenal (*n* = 17)	HRS (*n* = 14)	ATN (*n* = 22)	*P*
Serum creatinine (mg/dL)	2.79 ± 0.8	1.76 ± 0.2	2.21 ± 0.79	2.7 ± 1.23	0.003
Sodium (mEq/L)	132 ± 6.71	132 ± 4.93	129 ± 5	133 ± 7.77	0.257
Potassium (mEq/L)	4.94 ± 0.8	4.39 ± 0.61	4.41 ± 0.955	4 ± 0.54	0.004
Glomerular filtration rate (mL/min/1.73 m^2^)	35.6 ± 20.5	53.2 ± 13.84	44.3 ± 22.8	45.98 ± 47.48	0.475
Mean arterial pressure (mmHg)	89.5 ± 11.49	77.2 ± 14.2	74.86 ± 9.17	69.78 ± 15.35	0.0001
Urine sodium (mEq/L)	29.2 ± 7.81	29.3 ± 13.18	12.4 ± 5.6	62.68 ± 8.17	0.0001
Urinary IL-18 (µg/g creatinine)	582.34 ± 98.24	451.47 ± 121.73	953.5 ± 273	1687.1 ± 447	0.0001
Urinary NGAL (µg/g creatinine)	232.63 ± 41.31	161.15 ± 60.75	380.6 ± 132.32	580.51 ± 238.75	0.0001
Fractional excretion of sodium (FeNa) (%)	0.32 ± 0.17	0.54 ± 0.24	0.15 ± 0.07	4.05 ± 1.05	0.0001

CKD: chronic kidney disease, HRS: hepatorenal syndrome, and ATN: acute tubular necrosis.

Significant at *P* < 0.05; *P* < 0.01; and *P* < 0.001.

**Table 3:** Simple linear regression of urinary IL-18 and urinary NGAL in renal impairment patients.

	Urinary NGAL (µg/g creatinine)		Urinary IL-18 (µg/g creatinine)	
	*β*(*r*)	*P*	*β*(*r*)	*P*
Serum albumin (g/L)	0.129	0.293	0.114	0.355
Serum creatinine (mg/dL)	0.465	<0.001	0.422	<0.001
Serum bilirubin(mg/dL)	0.1212	0.325	0.0502	0.668
Urinary IL-18 (µg/g creatinine)	0.9	<0.001		
Urinary NGAL (µg/g creatinine)			0.9	<0.001
Fractional excretion of sodium (%)	0.687	<0.001	0.807	<0.001
Mean arterial pressure (mmHg)	0.339	0.005	0.329	0.006

*β*: regression coefficient.
